# Energy storing and return prosthetic feet improve step length symmetry while preserving margins of stability in persons with transtibial amputation

**DOI:** 10.1186/s12984-018-0404-9

**Published:** 2018-09-05

**Authors:** Han Houdijk, Daphne Wezenberg, Laura Hak, Andrea Giovanni Cutti

**Affiliations:** 1 Department of Human Movement Sciences, Faculty of Behavioral and Movement Sciences, Vrije Universiteit Amsterdam, Van der Boechorststraat 9, 1081 BT Amsterdam, The Netherlands; 2 Department of Research and Development, Heliomare Rehabilitation, Wijk aan Zee, the Netherlands; 3 Department of Health & Technology | Human Kinetic Technology, The Hague University of Applied Sciences, The Hague, The Netherlands; 4 Production Directorate, Applied Research, INAIL Prosthesis Center, Vigorso di Budrio, Bologna, Italy

**Keywords:** Amputation, Prosthesis, Stability, Symmetry, Gait, Rehabilitation

## Abstract

**Background:**

Energy storing and return (ESAR) feet are generally preferred over solid ankle cushioned heel (SACH) feet by people with a lower limb amputation. While ESAR feet have been shown to have only limited effect on gait economy, other functional benefits should account for this preference. A simple biomechanical model suggests that enhanced gait stability and gait symmetry could prove to explain part of the difference in the subjective preference between both feet.

**Aim:**

To investigate whether increased push-off power with ESAR feet increases center of mass velocity at push off and enhance intact step length and step length symmetry while preserving the margin of stability during walking in people with a transtibial prosthesis.

**Methods:**

Fifteen people with a unilateral transtibial amputation walked with their prescribed ESAR foot and a SACH foot at a fixed walking speed (1.2 m/s) over a level walkway while kinematic and kinetic data were collected. Push-off work generated by the foot, center of mass velocity, step length, step length symmetry and backward margin of stability were assessed and compared between feet.

**Results:**

Push-off work was significantly higher when using the ESAR foot compared to the SACH foot. Simultaneously, center of mass velocity at toe-off was higher with ESAR compared to SACH, and intact step length and step length symmetry increased without reducing the backward margin of stability.

**Conclusion:**

Compared to the SACH foot, the ESAR foot allowed an improvement of step length symmetry while preserving the backward margin of stability at community ambulation speed. These benefits may possibly contribute to the subjective preference for ESAR feet in people with a lower limb amputation.

## Background

Energy storing and return prosthetic (ESAR) feet have been available for decades. These prosthetic feet include carbon fiber components, or other spring-like material, that allow storing of mechanical energy during stance and releasing this energy during push-off [1]. This property has long been claimed to reduce the metabolic energy required for walking and hence improve walking economy. However limited scientific evidence has been found to corroborate this hypothesis [2–7]. Biomechanical studies have demonstrated enhanced mechanical energy storage in early stance and a considerable increase in positive power during push-off while using ESAR feet compared to conventional rigid feet [8–11]. In addition, studies have demonstrated that the increased external mechanical work during prosthetic walking seems to depend on a reduced push-off power [12] and that this is mitigated when walking with ESAR feet [9, 13]. Nevertheless these effects on mechanical energy transfers during walking, do not clearly translate into positive effects on metabolic energy expenditure and gait economy [14, 15]. It has been suggested that positive effects of increased mechanical ankle push-off power, are negated by an increased muscle activation required for body support or to control power transfer across residual joints in the prosthetic leg [16–18].

Despite the apparent absence of increased walking economy, ESAR feet remain the feet of preference for most people using lower limb prostheses [19, 20]. This gives rise to the consideration that other functional benefits, beyond economy, should exist. It has previously been shown that ESAR feet could reduce mechanical load, and therefore potentially prevent overload injuries in prosthetic or intact leg [21]. Alternatively, recent insights in the gait pattern of people with a lower limb amputation suggest that the enhanced ankle push-off power with an ESAR foot might enhance gait stability and improve gait symmetry [22].

**Fig. 1:**
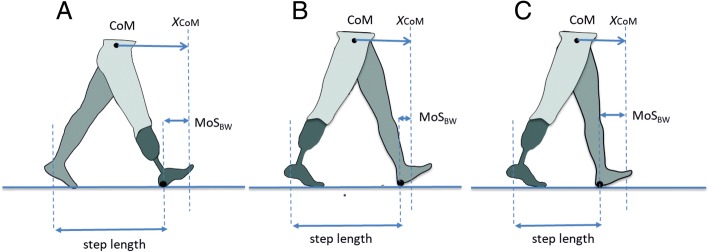
During walking, forward progression is maintained when the extrapolated center of mass (X_CoM_) projects anterior to the posterior border of the base of support at toe-off, i.e. when the backward margin of stability (MoS_BW_) at toe off is positive. In prosthetic gait, control of MoS_BW_ is affected by reduced push-off power of the prosthetic foot. **a** Depicts the prosthetic step for which no problem occurs. **b** Depicts the intact step. Due to the reduced push-off power of the prosthetic foot the center of mass velocity is reduced and hence X_CoM_ projects less far anteriorly. With normal step length, the MoS_BW_ would be reduced causing a treat for a loss of progression or a backward fall. **c** When the intact leg step length is reduced, MoS_BW_ is restored but at the expense of step length asymmetry (i.e intact step is shorter compared to the prosthetic step)

A stable gait requires that the body’s center of mass is controlled relative to the continuously changing base of support, i.e. the stance foot. In the fore-aft direction, this entails that the body’s center of mass needs to pass the leading foot during each stance phase, otherwise an interrupted forward progression or backward fall will occur [23]. The likelihood for the center of mass to successfully pass the leading foot can be assessed using the ‘margin of stability concept’ postulated by Hof et al. [24, 25]. Based on the inverted pendulum characteristics of human gait the position of the center of mass over time can be predicted based on its initial position, its velocity and the natural frequency of the inverted pendulum. Using these parameters, the so-called extrapolated center of mass can be calculated (Fig. 1). To maintain forward progression and make a subsequent step, the extrapolated center of mass needs to project anterior to the posterior border of the leading foot at the instant of toe-off of the trailing leg. The distance between the extrapolated center of mass and posterior border of the foot indicates the backward margin of stability. The smaller the backward margin of stability, the bigger the chance that the center of mass will not pass the foot in the presence of perturbations during single leg stance.

Recently, we have [22] demonstrated the effect of a reduced ankle push-off power on regulating the backward margin of stability during the intact step in people with a lower limb prosthesis. It was shown that due to a reduced ankle push-off power the center of mass velocity at toe-off of the prosthetic leg is lower compared to the contralateral step. This results in a reduction in the forward projection of the extrapolated center of mass and hence a potentially reduced backward margin of stability. To preserve sufficient backward margin of stability, people walking with a lower limb prosthesis appear to reduce intact leg step length, even though this inevitably leads to step length asymmetry (Fig. 1). From this mechanism, it can be derived that a prosthetic foot and ankle that increases push-off power might be beneficial as it would allow the user to improve gait symmetry without reducing the backward margin of stability.

In this study, we investigated the potential effect of energy storing and return feet on gait symmetry and backward margins of stability in a group of people with a transtibial amputation. We compared level ground walking using an ESAR foot and a SACH foot and hypothesized that the higher push-off power of the ESAR foot compared to the SACH foot will increase center of mass velocity at toe-off, increase intact step length and step length symmetry without reducing the backward margins of stability.

## Methods

Data used for this study was previously collected and published to assess differences in external work during walking with ESAR and SACH feet [9]. The specific details on data collection and analysis relevant for the current study are outlined below.

### Participants

Fifteen male participants with a transtibial prosthesis (age 55.8 ± 11.1 yr., weight 86.0 ± 12.6 kg, height 1.74 ± 0.04 m) were included in this study. All participants underwent amputation due to trauma, were classified at K3 level, and were free from other musculoskeletal, neurological or cardiovascular co-morbidities. All participants had walked with an ESAR prosthetic foot for at least two years before inclusion in the study. They were all informed on the study aim and procedure and provided written informed consent. The study was approved by the INAIL research board (Commissione Tecnico Scientifica, Budrio, Italy), and performed in accordance with the declaration of Helsinki.

### Procedure

Participants visited the prosthetic center on two separate days to assess their gait pattern while using their prescribed ESAR foot (for all participants this was the Vari-Flex, Össur, Iceland) and a SACH foot (1D10, Ottobock, Germany). On the first day gait analysis was performed while participants walked with their prescribed ESAR foot to which they were already accustomed. At the end of this measurement session participants were fitted with the SACH foot below their existing socket, which was aligned by a certified prosthetist. Participants used this foot in their daily life during the next 24 h to get accustomed to it, before returning to the clinic for a subsequent gait analysis using this SACH foot.

During the gait analysis participants walked up and down a 10-m walkway at a fixed walking speed of 1.2 m∙s^− 1^, which was controlled online using photocells (Microgate RaceTime2, Italy). A fixed speed over all participants and conditions was selected, since the outcome measures of this study are highly affected by walking speed, and potential speed differences between conditions would obscure the direct dependence of the analyzed gait parameters on foot type. The speed of 1.2 m∙s^− 1^ was selected as it was expected that participants would be able to walk comfortably at this speed with both types of feet. Self-selected walking speed of the participants was measured before the experiment with the ESAR foot as a reference. This self-selected speed appeared on average to be slightly but significantly higher (1.27 m∙s^− 1^, *p* = 0.03). Data from a minimum of 3 strides were collected for both intact and prosthetic leg while walking with the two different prosthetic feet.

### Data collection

Kinematic data was collected using a 10-camera opto-electronic system at 100 Hz (VICON; Oxford, United Kingdom). Markers were attached bilaterally on the anterior and posterior iliac spines, lateral epicondyles of the femur, lateral malleolus of the fibula. For the prosthetic side, lateral malleolus location was approximated as the distal end of the rigid pylon. Ground reaction forces were measured at 1000 Hz using two force plates (0.60 × 0.40 m. Kistler: Winterthur, Switzerland) embedded in the middle of the walkway. Gait speed while crossing the force plates was monitored using two photocells (Microgate Racetime 2; Bolzano, Italy).

### Data analysis

Force plate data was filtered at 100 Hz using a fourth order zero lag Butterworth low pass filter. All analyses were performed in the sagittal plane of progression. Force plate data was used to identify initial contact and toe-off based on a threshold vertical force of 25 N. Prosthetic step length (SL_prosthetic_) was calculated as the distance between the malleolus marker of the prosthetic leading and intact trailing leg at the moment of initial contact. Intact step length (SL_intact_) was calculated in a similar method at the time of initial contact of the intact leg. Step length symmetry (SL_symm_) was defined as the difference between prosthetic step length and intact step length:

1 $$ {SL}_{symm}={SL}_{prosthetic}-{SL}_{intact} $$

Power generated by the prosthetic foot and ankle during stance was calculated using the method outlined by Prince et al. [8, 26], summing both the translational power and rotational power transferred from the foot to the shank:

2 $$ {P}_{ankle}={\boldsymbol{F}}_{dist}\bullet {\boldsymbol{v}}_{dist}+{\boldsymbol{M}}_{dist}\bullet {\boldsymbol{\omega}}_{shank} $$

where the subscript ‘*dist’* represents the distal point of the rigid part of the prosthetic leg at approximately the level of the malleoli of the intact leg. ***F***_*dist*_ and ***v***_*dist*_ are the reaction forces and linear velocity of this distal point, ***M***_*dist*_ represent the net moment at the distal point and ***ω***_*shank*_ the angular velocity of the shank. Ankle push-off work (W_ankle_, J∙kg^− 1^) was determined as the time integral of the positive power burst prior to toe-off.

Center of mass position (CoM) was calculated from the average of the four iliac markers. Center of mass velocity (v_CoM_) was calculated as the time derivative of the CoM position. Following the description of Hof et al. (2005, 2008) the extrapolated center of mass (X_CoM_), represents the predicted position of the center of mass after the natural cycle time of the pendular motion of the leg, and was calculated as:

3 $$ {X}_{CoM}= CoM+{v}_{CoM}\sqrt{\raisebox{1ex}{$l$}\!\left/ \!\raisebox{-1ex}{$g$}\right.} $$

with *l* representing leg length (distance from floor to trochanter major), *g* representing gravitational acceleration and $$ \sqrt{\raisebox{1ex}{$l$}\!\left/ \!\raisebox{-1ex}{$g$}\right.} $$ representing the natural frequency of the leg pendulum.

The backward margin of stability was defined according to Hak et al. [23]:

4 $$ {MoS}_{BW}={X}_{CoM}- BoS $$

with the posterior border of the base of support (BoS) of the leading leg represented by the malleolus. Hence, a positive MoS_BW_ indicates a stable condition in which the CoM passes the leading stance foot. This definition is in line with previous studies of Hak et al. [22, 27]. However, it is the reverse from the original definition of Hof et al. [25], which was postulated for upright standing during which X_CoM_ should not pass the border of the base of support. Therefore, contrary to the current definition, Hof et al. defined MoS positive when it did not exceed the border of the BoS.

All primary outcomes were assessed at the instant of toe-off of the trailing prosthetic leg, as this is the instant that the trailing leg can no longer generate push-off power to accelerate CoM. Hence, at toe-off the condition for dynamic stability (i.e MoS_BW_ > 0) needs to be satisfied. However, v_CoM_ and MoS_BW_ were also analyzed at heel strike of the intact leading leg (occurring prior to toe-off), to test whether differences in these parameters between feet originate primarily during the double support phase, during which push off power is predominantly generated.

Except from step length and step length symmetry, outcomes were only analyzed for the step in which the prosthetic leg is the trailing push-off leg and the intact leg is the leading leg (i.e. the intact step). All parameters were separately analyzed for each of the three strides collected, after which outcomes were averaged to obtain a mean score for each subject and prosthetic foot type.

### Statistics

The differences in push-off work of the prosthetic foot, step length, step length symmetry, v_CoM_ and MoS_BW_ at toe-off between walking with ESAR and SACH foot were analyzed using paired samples t-tests. Differences in the changes in v_CoM_ and MoS_BW_ during double support, from heel contact to toe-off, between ESAR and SACH were analyzed using two-way ANOVA. Significance level was set a-priori at *p*-value < 0.05.

## Results

All participants succeeded to walk comfortably on both ESAR and SACH foot. Walking speed in both foot conditions was on average 1.22 ± 0.02 m∙s^− 1^. Stride length did not differ between condition (1.38 ± 0.06 vs. 1.37 ± 0.07 for ESAR vs. SACH).

**Fig. 2:**
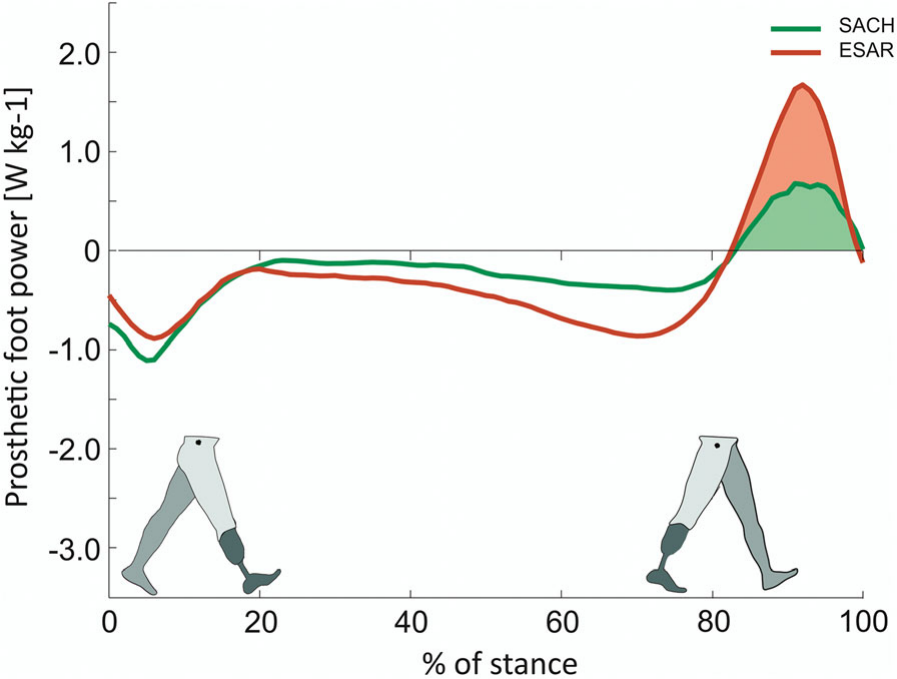
Push-off power of the prosthetic foot as a function of normalized stance time. The ESAR foot (red) generates negative power, storing elastic energy, in midstance and generates a higher positive push-off power, returning, more elastic energy during push-off compared to the SACH foot (green). The coloured surface below the power profile indicates the amount of work delivered during push-off (Figure amended from Wezenberg et al. 2014 [9])

**Fig. 3:**
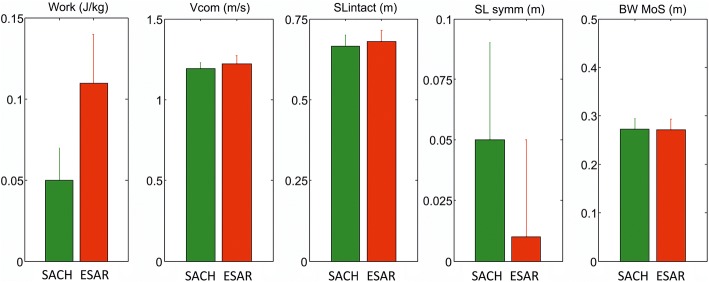
Difference in push-off work of the prosthetic foot (Work), center of mass velocity (v_com_), intact step length (SLintact), step length symmetry (SLsymm) and backward margin of stability (MOS_BW_) between walking with the SACH foot (green) and ESAR foot (red). * denotes significant difference between foot conditions

Push-off power of the prosthetic foot was significantly higher while walking with the an ESAR foot compared to a SACH foot (Fig. 2). This resulted in an increase of push-off work of 120% when walking with the ESAR foot compared to SACH (0.11 ± 0.03 vs. 0.05 ± 0.02 J∙kg^− 1^ for ESAR and SACH resp., *p* < 0.001)(Fig. 3), as was previously reported by Wezenberg et al. [9].

Step length of the intact step was larger when walking with the ESAR foot compared to the SACH foot (0.68 ± 0.03 vs. 0.66 ± 0.04 m, *p* = 0.004) (Fig. 3). This increase in intact step length improved step length symmetry (*p* < 0.001). The difference in step length between the intact and prosthetic step, reduced from 0.05 ± 0.04 m while walking with the SACH foot, to only 0.01 ± 0.04 m while walking with the ESAR foot.

**Fig. 4:**
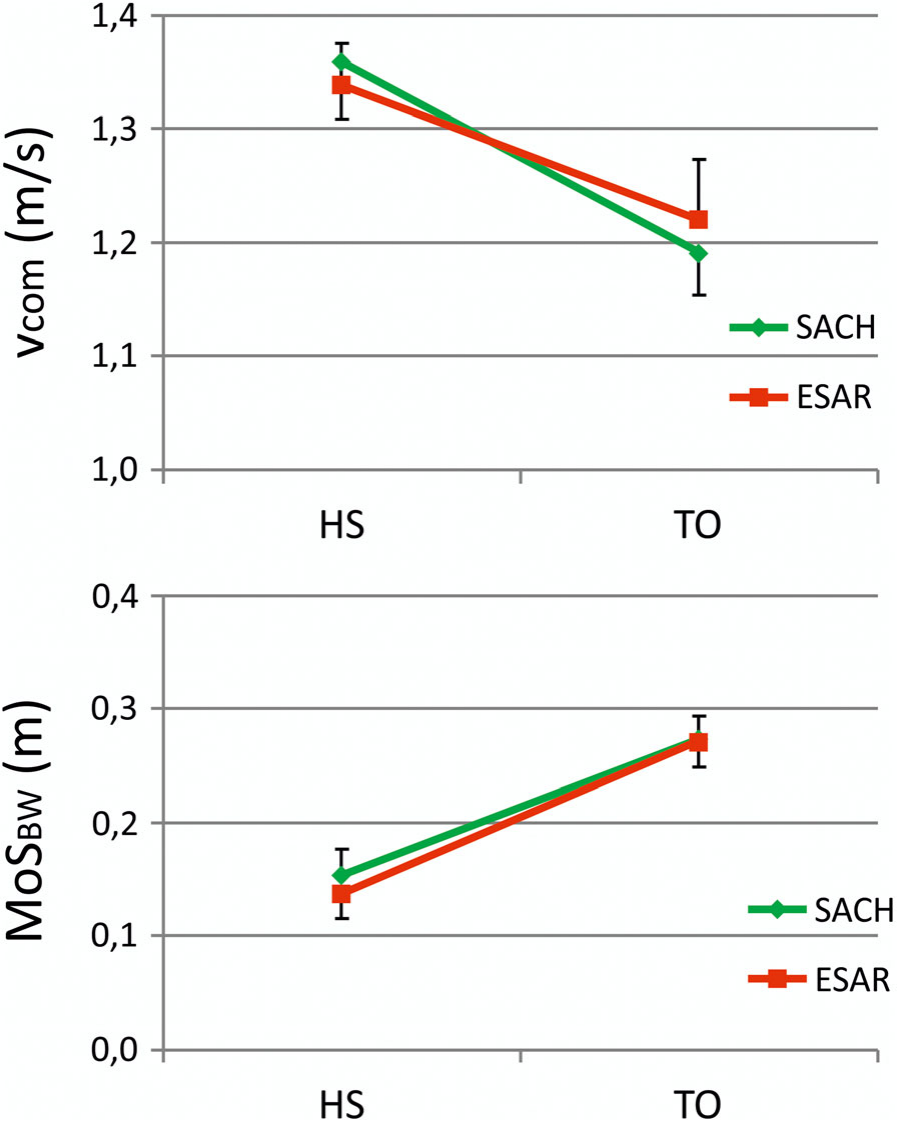
Change in center of mass velocity (v_com_) and backward margin of stability (MoS_BW_) during the double support phase, from heel strike (HS) of the intact leg until toe-off (TO) of the prosthetic leg during walking with the SACH (green) and ESAR (red) foot

Center of mass velocity decreased significantly more during the double support phase when walking with the SACH foot compared to walking with the ESAR foot (interaction effect foot x time *p* < 0.000) (Fig. 4). At the instant of toe-off, center of mass velocity was higher when walking with the ESAR foot compared to the SACH foot (1.22 ± 0.05 vs 1.19 ± 0.04 m∙s^− 1^, *p* = 0.03, Fig. 3), while there was no difference at heel strike.

Concurrently, backward margin of stability was lower at heel strike when walking with ESAR compared to SACH (0.137 ± 0.022 vs 0.153 ± 0.023 m, *p* = 0.001), but showed a larger increase during double support (interaction effect foot x time p = 0.001) (Fig. 4). Hence, at toe off the backward margin of stability did not differ significantly between foot conditions (0.271 ± 0.022 vs 0.272 ± 0.022 m, *p* = 0.36) (Fig. 3).

## Discussion

In this study, we investigated a potential functional benefit of energy storing and return (ESAR) prosthetic feet. Specifically, we investigated whether ESAR feet could preserve gait stability while restoring gait symmetry. Such benefit may possibly contribute to the general preference of people with a lower limb amputation for these types of prosthetic feet, considering the previously observed absence of improvements in gait economy. Both gait stability and symmetry are frequently mentioned objectives for people with a lower limb amputation who need to regain walking ability with a prosthesis.

Based on a simple biomechanical model of human gait (Fig. 1) and the known increase in push-off power, we hypothesized that compared to the conventional SACH feet ESAR feet would increase center of mass velocity at toe-off, increase extrapolated center of mass forward projection and as such potentially enhance the backward margin of stability. This would allow the prosthetic user to increase intact step length and restore step length symmetry, without reducing the backward stability margin.

Conditional for this hypothesis is an increase in push-off power of ESAR feet relative to SACH feet. Indeed for the type of ESAR foot used in this study the positive work performed by the foot and ankle unit was on average 2.2 times higher compared to work performed by the SACH foot, but remains about half of the intact leg ankle foot and ankle power [9]. Similar results have been found in previous studies into the energy return of ESAR and SACH feet [28, 29], although the magnitude of energy return of prosthetic feet reported in literature varies considerably. This is not only due to differences between prosthetic feet type, but also due to methodological differences. Specifically, the way researchers deal with the non-rigidity of the foot segment and the lack of a well-defined ankle center of rotation in the calculation of power generated by the compliant prosthetic foot and ankle system [8, 30]. These issues obscure the comparison of energetic properties between different feet presented in literature.

The increased push-off power of the ESAR foot likely results in a higher velocity of the center of mass at toe-off. In fact, center of mass velocity decreased during the double support phase for both prosthetic feet, but this decrease was attenuated more in the ESAR condition compared to the SACH condition. This difference in the change of center of mass velocity during the specific phase of double support indicates that increased push-off power with ESAR likely accounts for the observed effects, as push off power is predominantly generated during this double support phase. This relation between ankle push-off power and center of mass propulsion is corroborated by previous simulation studies, which mathematically showed a direct relation between the amount of energy return of prosthetic feet and the propulsion of the body’s center of mass [31, 32]. Alternatively, it might be argued that the difference in roll-over shape [33] between the two types feet, might contribute to the difference in center of mass velocity at toe off. It has been demonstrated previously that the roll-over shape of the ESAR foot used in this study has a larger arc length, providing a longer lever to the foot segment [9]. In passive feet a proper roll-over shape could also enhance the step-to-step transition, apart from ankle push off power, and attenuate deceleration of the center of mass double support [34]. It is however not known how roll-over shape and push off power interact in dynamic ESAR feet, and how the effect on center of mass velocity could be partitioned over both. This should be explored in the future studies.

Because of the larger center of mass velocity at toe-off with the ESAR foot, the extrapolated center of mass projects more anterior compared to the SACH condition. This allows the prosthetic user to make a larger step with the intact leg, without compromising the backward margin of stability. This increase in intact step length, observed with the ESAR foot, resulted in increased step length symmetry when walking with the ESAR foot. Although step length symmetry, on its own, is not necessarily a functional benefit [22], often gait symmetry is considered a goal in gait training [35, 36]. From a cosmetic point of view patients might prefer a close to normal symmetric gait pattern as to not stand out in the crowd. Furthermore, it has been speculated that gait symmetry might indirectly provide functional benefits as it could reduce mechanical overload on the intact and residual leg and on the low back, which are both common co-morbidities in people with a lower limb amputation [21, 37]. This difference in mechanical loading of the intact leg has indeed been presented previously (in terms of external mechanical work) for this data set [9]. Hence, improving gait symmetry might be considered a relevant functional benefit of ESAR feet.

The backward margin of stability at toe off was similar between foot condition. Nevertheless, this can be interpreted as a positive effect of the ESAR foot. Without the additional push off power of the ESAR foot, as in the SACH foot, an increased intact step length would have resulted in reduced margins of stability given the constraints outlined in Fig. 1. This can further be substantiated when analyzing the change in backward margin of stability during double support (Fig. 4), the phase during which push-off occurs. At heel strike of the intact leg margin of stability is smaller in the ESAR condition compared to the SACH condition. This is due to the fact that participants walk with a larger intact step length with the ESAR foot, while center off mass velocity at heel strike is similar between conditions. During double support the center of mass velocity decreases in both conditions but this decrease is smaller with the ESAR foot. The concomitant higher center of mass velocity at toe off with the ESAR foot results in similar margins of stability between feet, making up for the initial negative effect of increased step length. A similar effect of ankle push-off on the control of the backward margin of stability can also be seen when comparing the intact and prosthetic step in prosthetic gait [22]. Such enhanced control over the backward margin of stability might affect balance confidence of the prosthetic user, which has been indicated as an important predictor of self-reported mobility performance and social activity [38, 39]. When the prosthetic user is perceptive to this change in control over the backward margin of stability, this effect might contribute to the preference of many users for energy storing and return prosthetic feet over solid feet.

This study, designed to investigate a specific mechanical consequence of the constraints of prosthetic feet, is subject to several limitations. The experiments were performed at a fixed walking speed between conditions (prosthetic foot). This was done to avoid the confounding effect of walking speed on the outcome parameters, which would obscure interpretation of the underlying mechanics. Although strong evidence is lacking [15], it has been suggested that people tend to walk slower with SACH feet compared to ESAR feet. Such a reduction in walking speed could be a strategy to cope with the indicated constraint on gait stability. While in this study we demonstrate that this specific constraint of SACH feet on step length symmetry and margin of stability exists at equivalent speeds, it should be explored in the future how differences in walking speed influence this constraint. A second limitation is the fact that we included participants that were all currently using the ESAR Variflex foot. The SACH foot was provided to them for the purpose of this experiment. They were allowed a 24-h accommodation period at home to get used to the SACH foot. Moreover, they all had some previous experience with SACH and most of them use a SACH foot in their current bathing/in-house prosthesis. Nevertheless, we cannot rule out if a lack of acclimation on the SACH foot influenced our results. However, the effects found in our study agreed very well with the hypothesized differences between feet. This provides some confidence to the fact that hypothesized mechanical constraints indeed exist in prosthetic walking, although with more practice people might find smart strategies to cope with these constraints. Next to the effect of practice other factors might have influence the observed effects. Imperfect socket fit or alignment could affect step length and symmetry. We tried to minimize this effect by allowing participants to use their own socket with both feet, and by having a certified prosthetist optimize alignment of the SACH foot before the experiment. No participant indicated stump problems during the experiments. Another limitation of this study is that we only investigated one type of ESAR feet, i.e. Variflex (Össur, Iceland). In general, all ESAR feet do provide increased push power and are expected to allow for the improved control over the backward margin of stability and step length symmetry as found in this study. However, this general effect should be confirmed in other feet. Moreover, assessing these parameters could be used as a benchmark test for different prosthetic feet. Finally, results of this study were obtained in a group of relatively active persons with a transtibial amputation as a result of trauma. Generalization of the results to less active persons or different amputation causes or levels should be done with caution. For instance, within the population of people with transfemoral prosthesis, step length asymmetry is less consistently directed towards a shorter intact step length [36, 40]. Given the limitations presented above, generalization of the results to less controlled conditions and their contribution to the experienced benefits of ESAR feet in daily life should be interpreted with care. However, we believe that this study design does reflect a basic mechanical constraint of prosthetic feet and the related effect on step length symmetry and margin of stability.

## Conclusion

In conclusion, this study showed that the energy storing and return (ESAR) prosthetic foot can enhance center of mass propulsion, thereby allowing a symmetric gait pattern while preserving the backward margin of stability. These benefits on gait stability and symmetry might possibly contribute to the general preference of people with a transtibial amputation for these dynamic prosthetic feet. Current findings can prove to be helpful in the design, prescription and evaluation of future prosthetic feet.
